# Individual quality and environmental factors interact to shape reproduction and survival in a resident bird of prey

**DOI:** 10.1098/rsos.231934

**Published:** 2024-09-11

**Authors:** Roman Bühler, Thomas V. Riecke, Kim Schalcher, Alexandre Roulin, Bettina Almasi

**Affiliations:** ^1^ Swiss Ornithological Institute, Seerose 1, Sempach CH-6204, Switzerland; ^2^ Department of Ecology and Evolution, University of Lausanne, Building Biophore, Lausanne CH-1015, Switzerland; ^3^ Wildlife Biology Program, University of Montana, Missoula MT 59812, USA

**Keywords:** fitness, survival, path analysis, barn owl, sex-specific reproductive costs

## Abstract

Investigating among-individual differences in reproductive success and survival is essential for understanding eco-evolutionary processes. We used 5 years of demographic data from 556 breeding barn owls (*Tyto alba*) to estimate associations between intrinsic and extrinsic covariates on survival and reproduction throughout the annual cycle. As males and females have distinct roles in reproduction, environmental conditions and individual quality may be differentially linked to their fitness at different time points. Males breeding early and inhabiting prey-rich areas experienced higher reproductive success but faced greater reproductive costs. Indeed, the number of offspring a male cared for was negatively associated with his body condition and survival. However, our results indicate that these influences can be mitigated in males experiencing favourable post-breeding environmental conditions. For female owls, early breeding and high food availability during the breeding period were linked with increased reproductive success. Prey availability during incubation and higher reproductive output were associated with higher survival into the next breeding period in females. Unlike males, females did not exhibit obvious trade-offs between reproductive success and survival. Our research demonstrates trade-offs between fecundity and survival, and that females paired with males able to provide sufficient food experience higher survival and reproduction.

## 1. Introduction

Investigating variation in reproductive success and survival is essential for understanding eco-evolutionary processes and population dynamics [1,2]. Rearing offspring is one of the most energetically costly activities for adult birds [2,3], and depending on their parental role, the consequences of reproduction and the trade-off between reproductive effort and survival may differ for males and females [4].

Both intrinsic (individual-specific, e.g. age, phenotype) and extrinsic (environment-specific, e.g. resource availability, meteorological conditions) factors influence individual fitness. Food availability, in particular, can directly influence individual fitness and the timing of the annual life cycle. During the non-breeding period, food availability plays a critical role in determining the fitness of animals, as they need to meet their energy requirements for survival [5,6] and accumulate resources in preparation for the upcoming breeding investment [7,8]. Food availability before breeding can influence the timing of breeding, by influencing arrival time at the breeding grounds [9,10] and the timing of brood initiation [7,11,12]. The availability of food can influence initial investment via clutch size [12–15] as well as reproductive success [16–18]. Energy derived from resources is limited and must be allocated to different activities during the annual life cycle [2,3]. Consequently, a high energy investment in reproduction for a given breeding attempt is expected to trade off with survival or with subsequent breeding attempts within or among years [19–24]. For example, the decision to reproduce twice in the same year may result in reduced survival or reproductive success in the following year [20,23,24]. In addition, high investment in the first reproductive attempt may reduce the success of the second reproductive attempt [20,21]. High reproductive investments during one year may even cause animals to skip reproduction entirely the following year [19,22,25]. After reproduction the energetic investment needs to be recuperated, and food availability during the post-reproductive period and during winter can be crucial for survival [24]. Therefore, food availability can influence individual fitness either directly through survival perspectives or indirectly via reproductive costs.

Individual responses to extrinsic factors are also dependent on intrinsic factors [20,26–28]. Indeed, it is often observed that some individuals consistently outperform others, e.g. individuals with a higher breeding success also have a higher survival in any environmental context [29–31]. These variations in individual performance are generally rooted in intrinsic differences which are shaping their life-history strategies [2]. The most well-known intrinsic factors that can lead to different responses (i.e. life-history trade-offs) to the same environmental stressors are sex and age [2]. Older and therefore more experienced individuals seem to have a general advantage over younger individuals, showing higher survival and reproductive success [23,32,33]. Sex differences in physiology or behaviour can lead to sex-specific effects of environmental conditions on reproductive success as well as to sex-specific costs of reproduction [4,34,35]. However, there are other measurable intrinsic factors that correlate with individual quality. For example, genetically determined melanin-based coloration is often correlated to fitness measures which may lead to alternative life-history strategies [36,37]. In the European barn owl (*Tyto alba*), females with large melanic spots on their plumage (which has a genetic basis, [38]) breed at a younger age, have higher survival rates [39] and increased lifetime reproductive success [40].

Another measurable intrinsic factor that affects individual fitness is how individuals react under environmental pressure. Glucocorticoid hormones (GCs) are important mediators between an organism and its environment, enabling appropriate physiological and behavioural responses to environmental perturbations. Basal GC levels are responsible for the maintenance of energy homeostasis in response to energetic demands [41,42]. GCs are also part of the adrenocortical stress response, which controls the reallocation of resources to physiological functions important for self-preservation and survival when the environment becomes unpredictably challenging. GCs play an essential role in mediating trade-offs between different life-history traits that may affect different fitness components, as has been shown in barn owls and other bird species [43–46].

Identifying the factors that influence individual fitness requires detailed data on both individual performance and environmental conditions. The development and miniaturization of tracking devices makes it possible to equip small animals and to track them over long periods of time, determine their time of death and locate their breeding sites [47]. Combined with traditional capture–recapture and breeding success data, this allows a detailed assessment of the factors influencing individual fitness in a species with biparental care. In the present study, we investigated how prey availability and intrinsic factors are related to breeding success and annual survival. Prey availability varies greatly throughout the year [48], and a key aim of the study is to investigate at what point in the annual cycle prey availability most strongly influences annual breeding success and survival, and whether these associations differ between sexes. A secondary aim is to investigate whether breeding success imposes reproductive costs on survival, and whether these costs are similar for males and females.

We used a hierarchical modelling approach that integrates live–dead encounter data and fecundity information. This method enables us to assess both direct and indirect links between intrinsic and extrinsic factors on the survival and reproductive success of barn owls [49]. As the barn owl is an income breeder, we expect the onset of reproduction and the annual number of eggs to depend on prey availability during the early breeding period, i.e. pre-laying and incubation period [50,51], while the annual number of nestlings and fledglings is likely to depend mainly on environmental conditions during the brood-rearing period. Due to the different parental roles during reproduction [50,52,53], we expect that food availability at different times in the annual cycle will affect reproductive performance and adult survival depending on sex, and that the sexes will experience different reproductive costs. In addition to prey availability, we expect intrinsic factors such as age and individual quality to be associated with survival and reproduction. With respect to age, we expect a positive effect of experience on survival and reproduction [23,32,33]. In barn owls, annual survival is known to be strongly dependent on winter severity, with cold temperatures increasing energy requirements and persistent snow cover reducing prey accessibility [6]. We expect that individuals with lower reproductive investment will enter winter in better condition and will experience lower mortality. However, this disadvantage may be offset by high food availability during winter, allowing individuals to make high reproductive investment and experience high survival at the same time.

## 2. Material and methods

### 2.1. Study area

We studied barn owls in western Switzerland (46°49' N, 06°56' E) in an area of approximately 1000 km^2^ characterized by intensive agriculture and interspersed with villages and forests. The area can be divided into four geographical regions (plain of Orbe, plain of the Broye, Haut-Fribourg, Gros de Vaud) slightly differing in terms of topography and land use [48]. More than 400 nest boxes for barn owls have been installed throughout the study area since 1985.

### 2.2. Individual data

The breeding season of barn owls extends from February to October [54] and is followed by a resident wintering period. As soon as a breeding attempt was detected during the nest box controls, which took place every 4 weeks from February to September, standard monitoring was initiated with four visits per brood: a first visit just before hatching to determine clutch size, second and third visits when the oldest nestling were 25 and 35 days old, and a final visit when the oldest nestling was close to fledging, around 55 days old. Breeding adults were captured and ringed at the nesting site and data on body mass, age, plumage traits and sex were collected. Females were captured one to three times (before hatching, 25 d, 35 d), while males were captured one or two times (25 d, 35 d). Owls were either captured by blocking the nest box entrance during the day (owls incubating/roosting inside the nest box), or at night by using sliding door traps. Sex was identified by the presence (female) or absence (male) of a brood patch. The age of each animal was determined from previous encounters or via primary and secondary moult patterns; this allowed us to discriminate between a bird in its second year of life (when there is no moult) or an older bird (which shows moulted feathers; [55]). Eumelanin spottiness of plumage was assessed by measuring spot diameter (to the nearest 0.1 mm) of 10 spots on the belly and the breast [56]. Body mass of breeding adults was measured at different time points: shortly before hatching, 25 days after hatching and 35 days after hatching. Clutch size, number of nestlings and fledglings were recorded for each breeding attempt. Number of nestlings refers to the number of juvenile birds alive at the visit 25 days after hatching and number of fledglings was defined as number of nestlings reaching an age of 55 days. Whenever possible, two blood samples per adult were taken to determine GC concentration (baseline: within 3 min of capturing event [57]; stress-induced: after 25 min of handling). GC concentration was analysed following procedures described in [58]. Depending on the concentration of the internal control and the year of analysis, intra-assay variation ranged from 5% to 18% (mean 11.4 ± 3.8%) and inter-assay variation from 11% to 28% (mean: 19.6 ± 5.7%).

The final dataset included 556 adult individuals and 840 breeding attempts (2017: 291 breeding attempts of 244 individuals, 2018: 132 of 125, 2019: 162 of 134, 2020: 255 of 212, mean: 1.16 attempts/individual and year) between 2017 and 2020. During the 4-year study period, 442 individuals bred once, 73 twice, 33 three times and seven individuals four times. One individual was discovered alive but did not reproduce during the study period. Out of the 556 individuals (257 males and 299 females), 237 individuals (130 males and 107 females) were equipped with a lightweight VHF transmitter (µTag, Swiss Ornithological Institute), combined with a GPS transmitter (Technosmart, Italy) [48]. GPS data were used in other studies [48] and are only referenced because the GPS device was integrated with the VHF tag. The attached GPS/VHS tag combination weighted less than 13 g (which is less than 5% of body mass, mean body mass before tagging: 297 g, range: 260–440 g). Tags were attached with spectra-tubular filament (4.7 mm, polyethylene, Bally Ribbon Mills, US) using a wing loop harness. The VHF transmitters were programmed to send signals during a single, synchronized week the following spring. During this time, we intensively searched inside the study area to check status of marked individuals (alive, dead or unknown/no signal detected). VHF-equipped birds were expected to have higher dead-recovery as well as higher recapture probability, as detection was not dependent on physically capturing alive individuals or finding dead birds by chance. All manipulations were performed under authorization from the Department of Consumer and Veterinary Affairs (VD and FR 2844 and 3213) and the Federal Office of Environment.

### 2.3. Prey availability around the nest box location

*Arvicola, Microtus* and *Apodemus* species are the staple prey of our study population of barn owls, although other small mammals are suitable prey as well but are much less common in the diet of barn owls [59,60]. Prey availability was monitored throughout the year using two indirect methods: fresh signs of voles (heaps, holes and runways of *Arvicola* sp. and *Microtus* sp.) were counted along 5 m × 1 m transects [61], and above-ground activity of all small mammal species was assessed by placing a plastic plate covered with a thin layer of graphite during two consecutive nights along each transect (adapted from [62]). The number of characteristic tracks of small mammals walking across the plates were then counted. Combining both methods, we obtained two indices for the activity density, one for voles and one for total small mammals. The two activity density indices were recorded in the four geographical regions of our study area, with four 9 km^2^ subplots selected in each region. Indices were counted six times per year at two-month intervals in four different habitat types: crop rotation (annual crop), grasslands (intensive and extensive grassland), border structures (1 m margin along roads) and biodiversity structures (hedges and wildflower areas). Nine transects were walked, and nine plates were laid in each habitat type during each counting period. This resulted in a total of 576 transects and an equal number of plates for each two-month period (details in [48]). To obtain estimates of the two activity indices on a continuous timescale for voles (transect method, hereafter vole activity density index) and small mammals (track-plate method, hereafter small mammal activity density index) separately, two generalized additive mixed models were fitted [48]. These indices will later be used to estimate prey availability within the different habitat types.

Both methods of assessing prey activity density have been shown to correlate well with estimates of relative abundance from live trapping [62,63]. In addition, vole populations in Central Europe have been shown to fluctuate synchronously across large spatial scales [64]. To estimate prey availability in the preferred hunting habitats [48,65], habitat composition within a radius of 1.5 km (average home range of adult barn owls in our study area; [48,58,65]) around each breeding site was calculated once in summer and once in winter (for details, see electronic supplementary material, S1), with the assumption that adult birds stay close to breeding site in winter [48]. Total prey activity density for all small mammals (hereafter ‘small mammal availability’) and voles (hereafter ‘vole availability’) in each of the four habitat types (crop rotation, grassland, biodiversity structures, border structures) was calculated by multiplying the activity density index of a habitat type at one of four different time points (pre-laying, breeding, rearing and winter) by the corresponding area.

## 3. Statistical analysis

**Figure 1.**
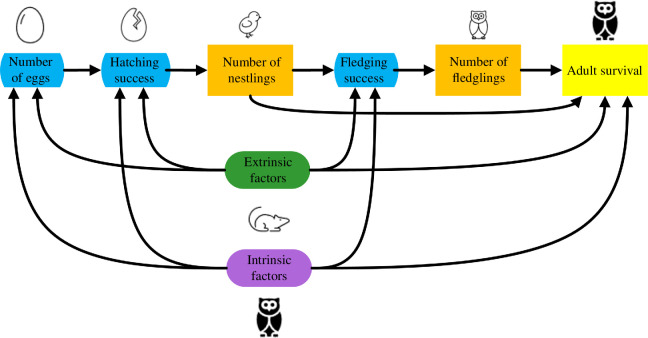
A simplified directed acyclic graph demonstrating the modelled relationships between intrinsic (purple) and extrinsic (green) covariates on various components of reproductive success (blue) and adult survival (yellow), as well as the relationships between reproductive investment and success (orange) on survival.

We estimated the effects of intrinsic (i.e. body condition, experience) and extrinsic (i.e. prey availability) covariates on the annual number of eggs laid, hatching success, fledging success and survival using a path analysis [49], which allows estimation of both direct and indirect effects as well as trade-offs between demographic components over the annual cycle (figure 1). As a specific example, increased prey availability may improve foraging, and consequently, body condition in barn owls. However, if increased prey availability leads to a major increase in reproductive investment, and if reproductive investment has a net negative effect on survival, then there may be no net effect of prey availability on survival.

Hereafter we will briefly introduce the covariates used; more details about the covariates can be found in the electronic supplementary material, S2. Experience was defined as 0 if individuals were first-time breeders or 1 if they had already attempted to reproduce at least once. Laying date was defined as the day of the year when the first egg of the first brood was laid. Body condition index was estimated for the first breeding attempt for incubation and brood-rearing separately by fitting a linear regression for each of the two time points using body mass as the response and wing length, time of the day of capture and sex as explanatory variables. The residuals of the linear regressions were then used as a proxy for body condition, thereby correcting for individual body size [66]. Since barn owls may breed twice if environmental conditions are favourable [54] and annual rather than single reproductive investments determine reproductive costs [3], we summed up the number of eggs, nestlings and fledglings for each adult and each year (electronic supplementary material, S4). Depending on the year, the proportion of individuals performing a second brood varied between 6% and 21% (females 9–24%, males: 2–18%). GC concentration and the diameter of plumage spots were included as measured proxies for individual quality [26,27,44,58,67–71]. Prey availability was calculated for the first reproductive event for the incubation and brood-rearing period, assuming that (i) prey availability during the first reproductive event affects the decision to produce a second brood and (ii) is correlated with prey availability later in the season.

As we model survival across each individual’s lifespan we have to deal with missing covariates because of imperfect detection (i.e. we do not observe every individual during every breeding season because some individuals skip reproduction, emigrate or die undetected), and because individuals survive beyond our observation process. Missing values for laying date, body condition, prey availability, corticosterone concentration and eumelanin spot diameter were estimated from a normal distribution with population-, individual- or sex-specific mean and standard deviation depending on the type of covariate (for details, see electronic supplementary material, S2). When measures of reproductive investment and success were not directly observed, they were estimated as a function of the previously described extrinsic and intrinsic covariates (see electronic supplementary material, S3 for more details).

### 3.1. Reproduction, recapture, recovery and survival estimates

As barn owls have very different reproductive roles, males and females were analysed separately. In a first model, we included mate experience as a proxy for mate quality, which may influence breeding success, to predict reproductive success and survival. Mate experience did not show a meaningful effect in any model and was therefore excluded from the final analyses. We are aware that mate quality may have a strong influence on reproductive success and subsequent survival, but as mate quality is not an easily measured parameter, it would need to be modelled in detail, which is beyond the scope of this study.

First, we modelled the number of eggs laid during each breeding season by each individual as a function of adult experience, laying date and pre-laying small mammal availability and vole availability using Poisson regression. Second, we modelled the annual number of nestlings reared by each individual as a binomial trial in which the number of nestlings was the outcome given the probability of hatching success and the number of eggs was the number of trials. Hatching success was modelled as a function of adult experience, the number of eggs laid, laying date, adult body condition measured during incubation and small mammal availability and vole availability during incubation.

We then modelled the annual number of fledglings produced by each individual as the outcome of a binomial trial given the number of nestlings, and the estimated fledging success. Like hatching success, fledging success was modelled as a function of adult experience, number of nestlings, laying date, adult body condition measured during brood-rearing and small mammal availability and vole availability during brood-rearing.

To estimate recapture (*p*), recovery (*r*) and survival (*φ*) probability, a mark–recapture–recovery model was used [72,73]. Survival probability was modelled as a function of sex and adult experience, adult body condition measured during brood-rearing, number of nestlings, number of fledglings, baseline GC concentration, stress-induced GC concentration and spot diameter of plumage. Due to the correlation between prey availabilities close in time, we decided to limit our analysis of the relationship between prey availability and survival on the periods of highest energy investment: winter and reproduction (represented by prey availability during incubation). We therefore included small mammal and vole availability in the linear predictor of survival during both the incubation period and winter. We modelled recapture probability and recovery probability separately for the ring-only and the GPS–VHF groups, as we were more likely to encounter individuals marked with active GPS–VHF transmitters. (See electronic supplementary material, S3, S5 and S6 for additional details.)

The model was fitted in JAGS [74] using the jagsUI package [75] in R 4.2.1 [76]. Before incorporating variables into the model, they were checked for correlation (correlation matrix, values less than 0.7), scaled and centred. We sampled 250 000 iterations with four chains with a burn-in of 100 000 iterations and retained every 25th iteration. We checked that $\hat{R}$ values of posterior distributions were less than 1.01, and visually inspected trace plots to check for convergence [77]. In the results, we report the median of the posterior distribution together with the 95% credible intervals (CrI), as well as the proportion of the posterior distribution on the same side of zero as the mean (*f*).

## 4. Results

### 4.1. Number of eggs

**Figure 2.**
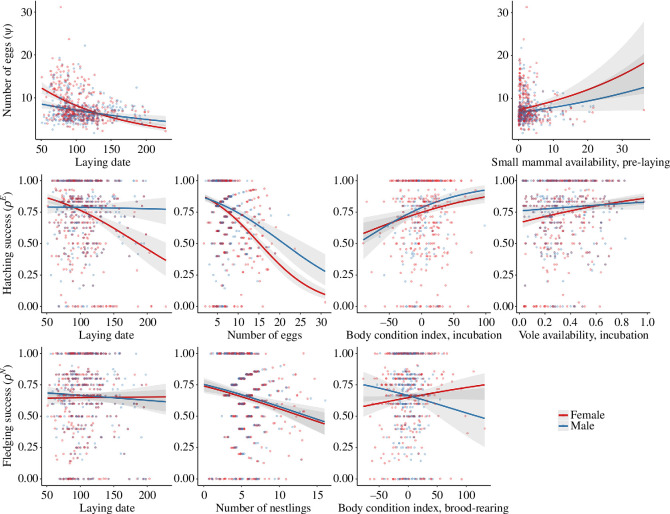
Mean estimates (solid lines) and 95% credible intervals (shaded area) of the relationship between annual number of eggs (ψ) , hatching success $\left(\rho^{E}\right)$ , fledging success $\left(\rho^{N}\right)$ and explanatory variables; from left to right: laying date, reproductive investment, body condition, prey availability. Females are presented in red and males in blue. Estimates, presented for experienced individuals based on the models presented in electronic supplementary material, S7, S8 and S9.

**Figure 3.**
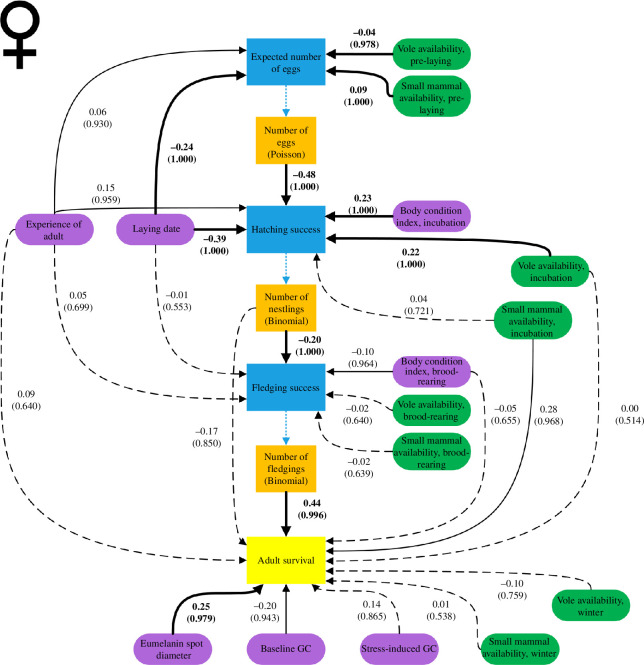
A directed acyclic graph demonstrating the relationships of intrinsic (purple) and extrinsic (green) covariates on female reproductive success (blue) and survival (yellow). Estimates and *f*-values (in brackets) are derived from the models shown in electronic supplementary material, S10 and are based on 556 individuals over 5 years. Bold arrows indicating *f*-values > 0.975, solid lines *f*-values between 0.9 and 0.974 and dashed lines *f*-values < 0.9.

Higher annual number of eggs were produced by females (figures 2 and 3, electronic supplementary material, S7) with earlier first clutches (*β* = −0.24, CrI: −0.29 to −0.18) and higher small mammal availability prior to laying (*β* = 0.09, CrI: 0.04 to 0.14). High vole availability prior to laying was linked to lower annual number of eggs produced (*β* = −0.04, CrI: −0.09 to 0.00). Experience of the female was weakly but positively related to the annual number of eggs (*β* = 0.06, CrI: −0.02 to 0.13).

**Figure 4.**
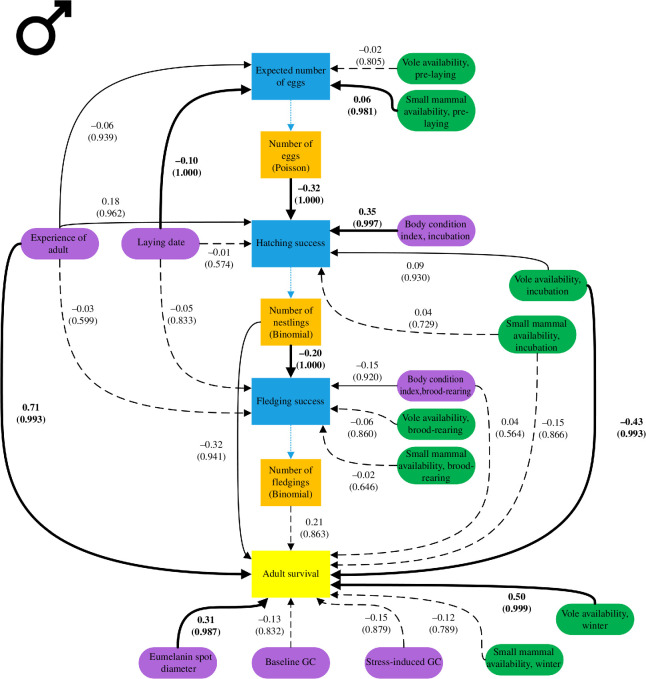
A directed acyclic graph demonstrating the relationships of intrinsic (purple) and extrinsic (green) covariates on male reproductive success (blue) and survival (yellow). Estimates and *f*-values (in brackets) are derived from the models shown in electronic supplementary material, S10 and are based on 556 individuals over 5 years. Bold arrows indicating *f*-values > 0.974, solid lines *f*-values between 0.9 and 0.974 and dashed lines *f*-values < 0.9.

Males showed a similar pattern with laying date and small mammal availability being associated with the annual number of eggs to which the male contributed (figures 2 and 4, electronic supplementary material, S7). Earlier first clutches (*β* = −0.10, CrI: −016 to −0.05) and increased small mammal availability (*β* = 0.06, CrI: 0.00 to 0.12) were related to a higher annual number of eggs. Contrary to the results found for females, vole availability was not strongly associated with annual number of eggs (*β* = −0.02, CrI: −0.07 to 0.03). In males, experience was linked with lower number of eggs per year (*β* = −0.06, CrI: −0.14 to 0.02).

### 4.2. Hatching success

In females, hatching success (figures 2 and 3, electronic supplementary material, S8) was positively related to high vole availability during incubation of the first clutch (*β* = 0.22, CrI: 0.12 to 0.32), early laying of the first clutch (*β* = −0.39, CrI: −0.53 to −0.26) and high female body condition index after laying the first clutch (*β* = 0.23, CrI: 0.13 to 0.32). High number of annually produced eggs was associated with lower hatching success (*β* = −0.48, CrI: −0.55 to −0.41) but positively associated with experience (*β* = 0.15, CrI: −0.02 to 0.32). However, small mammal availability was not related to hatching success.

In males, hatching success (figures 2 and 4, electronic supplementary material, S8) was positively linked with high male body condition after laying (*β* = 0.35, CrI: 0.12 to 0.53) and high vole availability during incubation (*β* = 0.09, CrI: −0.05 to 0.17). Experience was associated with higher overall hatching success (*β* = 0.18, CrI: −0.02 to 0.38) but was also linked with lower annual number of eggs (*β* = −0.32, CrI: −0.41 to −0.23). Small mammal availability and laying date did not seem to be related to hatching success.

### 4.3. Fledging success

In females, fledging success (figures 2 and 3, electronic supplementary material, S9) was negatively related to the number of nestlings (*β* = −0.20, CrI: −0.30 to −0.11) and positively associated with body condition during the brood-rearing period (*β* = 0.10, CrI: −0.01 to 0.20). No strong evidence was found for a relationship between fledging success and female experience, laying date or small mammal availability, as well as vole availability during the brood-rearing period.

In males, body condition during the brood-rearing period was positively related to fledging success (figures 2 and 4, electronic supplementary material, S9). Annual fledging success was negatively linked with body condition during the brood-rearing period of the first brood (*β* = −0.15, CrI: −0.35 to 0.06). Fledging success was negatively linked to the number of nestlings the male contributed to annually (*β* = −0.20, CrI: −0.31 to −0.10). Vole availability and small mammal availability during the brood-rearing period as well as laying date and experience of the male were not linked with fledging success.

### 4.4. Survival of adults

**Figure 5.**
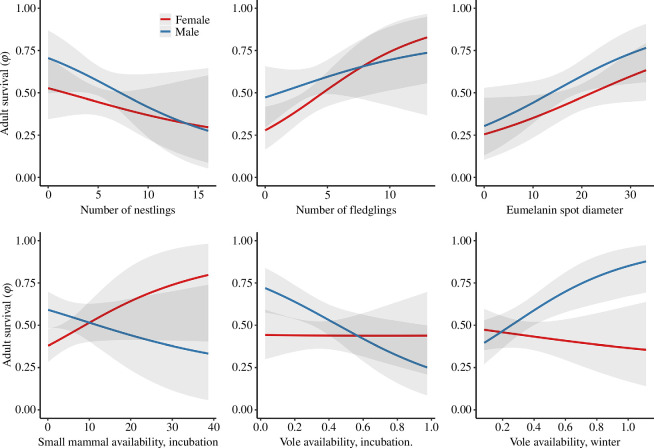
Mean estimates (solid lines) and 95% credible intervals (shaded area) of the relationship between adult survival (φ) and explanatory variables. Females are presented in red and males in blue. Estimates, presented for experienced individuals based on the model presented in electronic supplementary material, S10.

Female survival (figures 3 and 5, electronic supplementary material, S10) was positively related to annual number of fledglings produced (*β* = 0.44, CrI: 0.11 to 0.79). Furthermore, females with larger spot diameter on belly and breast were associated with higher survival compared with those with smaller spot diameters (*β* = 0.25, CrI: 0.01 to 0.50). There was a negative link of high baseline GC on female survival (*β* = −0.20, CrI: −0.46 to 0.04). Small mammal availability during incubation was positively related with female survival (*β* = 0.28, CrI: −0.02 to 0.60), while vole availability during incubation and small mammal availability and vole availability during winter were not linked with survival. We found no association between female survival and experience (electronic supplementary material, S6), body condition measured during the brood-rearing period, number of nestlings produced or stress-induced GC level.

Experienced males were associated with higher survival than non-experienced males (*β* = 0.71, CrI: 0.15 to 1.26; figures 4 and 5, electronic supplementary material, S6 and S10). Like females, the diameter of plumage spots was positively linked with male survival (*β* = 0.31, CrI: 0.04 to 0.60). For reproductive parameters, the strongest link with male survival was observed for the number of nestlings produced during the reproductive period (*β* = −0.32, CrI: −0.73 to 0.08). While vole availability during the incubation period was negatively associated with male survival (*β* = −0.43, CrI: −0.76 to −0.09), vole availability during winter was positively linked with survival (*β* = 0.50, CrI: 0.17 to 0.87). There was no support for a strong association between male survival and body condition during the brood-rearing period, number of fledglings, baseline and stress-induced GC, as well as small mammal availability during incubation and in winter.

## 5. Discussion

Males and females often have distinct roles in reproduction, which means that workloads may be greatest at different times during reproduction. Depending on sex and timing, environmental factors have different effects on fitness. Our study reveals that both environmental conditions and factors indicating individual quality can directly and indirectly affect adult survival. Depending on reproductive stage and sex, prey availability and individual quality factors were also differently associated with reproduction. Overall, males faced a trade-off between reproduction and survival, while female survival and reproduction were primarily influenced by female quality and environmental conditions. Below, we delve into how the examined intrinsic and extrinsic factors were related to reproductive performance and survival.

### 5.1. Extrinsic factors and reproduction and survival

In this study, we examined the impact of pre-breeding and breeding season food availability, as well as brood timing, on annual reproductive success and survival. High prey availability early in the breeding season (i.e. pre-laying and incubation) resulted in greater reproductive success for early-breeding males. However, the more offspring they reared and the more food they had, the lower their subsequent survival, suggesting reproductive costs. In contrast, females benefited from abundant prey early in the breeding season as they could lay more eggs throughout the reproductive period and achieve higher hatching success and subsequent survival. These different relationships between food availability and reproductive success and survival are probably due to the different roles of males and females in the reproductive system.

Egg production and incubation require significant energy expenditure from female birds [78–81] including barn owls, where females exclusively incubate [50,52]. Increased small mammal availability during incubation may therefore meet the females’ energetic needs, supporting egg production (which continues during the incubation period due to the asynchronous breeding), body condition maintenance and recovery from the investment, leading to enhanced female survival. Male barn owls are the only food provider for females and nestlings during incubation and early brood-rearing stages [52], as females do not contribute to food provisioning until late brood-rearing [53]. Male provisioning of food therefore increases the female’s nest attentiveness during incubation [82,83], positively influencing hatching success [78,84,85] and reducing the risk of early brood reduction [52]. High vole availability during incubation may therefore enable males to provide sufficient prey during this period, leading to improved hatching success and sustained female body condition.

The peak investment for males, however, is expected to happen during the brood-rearing phase when males are the main provider of food to the nestlings [53]. Because of these differences in peak investment in reproduction, it is likely that different environmental cues are used by females and males at different times to guide their investment in reproduction [86]. For female barn owls, food availability before egg-laying and during incubation was related to reproductive success and survival. In contrast, male reproductive success is mainly linked with food availability during brood-rearing. However, high investment during brood-rearing was negatively associated with male survival, particularly when prey was abundant during incubation. Prey availability during incubation seemed to influence male survival through two distinct pathways. Firstly, high vole availability during incubation of the first brood was positively associated with overall hatching success, leading to a subsequent increase in the number of nestlings, which is again negatively linked with male survival. Thus, we observed an indirect negative link of prey availability during incubation on male survival mediated by reproductive effort. Additionally, vole availability during incubation was directly negatively associated with survival. This suggests that males incur certain costs associated with high vole availability during incubation, not reflected in the number of nestlings. Higher prey availability earlier in the breeding season may lead to increased and/or early investment by males, such as energy-demanding courtship behaviour [87]. This investment may not be fully mirrored by the number of nestlings but would manifest as a direct negative link of vole availability during incubation on male survival. The extent to which the discussed costs influenced males seemed to depend on the environmental conditions experienced after the breeding season. High prey availability in winter (represented by high vole availability) may help males recover from the energy-demanding breeding period, therefore positively acting on survival [24].

Timing of the first clutch was linked with annual number of eggs the sexes contributed to, as well as with hatching success for females. Birds breeding early and under good conditions might either lay larger first clutches and/or have a higher probability to produce a second clutch. Birds in seasonal environments face time constraints and only birds reproducing early can start a successful second breeding attempt [54,88–90]. Abundant food resources at the onset of the breeding season generally facilitate earlier and more successful first breeding attempts, while also supplying the energy and time needed to initiate a potential second brood [91]. In barn owls, late and second clutches are usually larger than early and first clutches [60,65,92] suggesting that the observed link emerged from double brooding rather than from increased clutch size during first reproduction. Females exhibited a stronger association between laying date, the number of eggs and hatching success compared with males, potentially attributed to the nearly two times higher rate of second brood initiation in females (electronic supplementary material, S3). Because males primarily provide most of the food until the offspring achieve independence [53], their capacity to initiate a second brood is significantly constrained in terms of timing, unlike that of females. Females can leave their first brood before the offspring becomes independent and therefore have a greater chance of starting a second breeding attempt [54].

High food availability early in the breeding season may promote early laying, but whether birds engage in double brooding depends on food availability later in the season as well. Prey availability at the beginning of the breeding period, previous to laying, may be associated with prey availability later in the season. High small mammal availability is likely to reflect increased availability of mobile *Apodemus* sp. [93]. These prey species tend to move from forests and hedges into arable fields as the seasons progress [94], which are the preferred hunting areas for barn owls [95]. Consequently, early high small mammal availability can positively influence the first breeding attempt and increase the likelihood of a second breeding event, resulting in a higher number of eggs per breeding season. In fact, studies on female barn owls have demonstrated a correlation between the availability of *Apodemus* sp. in early-season food stores and the initiation of a second brood [88]. The connection between a decreasing annual number of eggs and high vole availability prior to egg-laying may have a similar underlying cause. Early high vole availability could indicate an initial peak in vole populations, which may seem advantageous at first. However, vole populations with high densities early in the year are susceptible to population crashes later in the season [96]. These crashes can have negative implications for the initiation of second broods, as resource availability later in the season becomes crucial for successful breeding attempts [91,97].

### 5.2. Intrinsic factors and reproduction and survival

We analysed the effects of experience of an individual (first-year breeders versus older individuals), their melanin-based coloration, body condition and the stress hormone GC on different demographic components. These four intrinsic quality measurements were positively linked with certain aspects of reproductive success and survival in both sexes. In males, a reproductive conflict arises between maximizing offspring numbers, which has a negative impact on body condition [98], and ensuring good survival, which is positively associated with body condition. Increasing current reproductive output at the cost of future fitness (survival and future reproduction) is an observed strategy in avian species [99–101]. Male body condition during incubation showed a positive association with hatching success, but there was a trade-off observed between self-maintenance and reproductive success [3,102]. Males in better body condition during incubation produced a larger number of nestlings, but this came at the cost of lower survival and decreasing body condition later in life. It appears that male barn owls sacrifice their body condition to improve their reproductive success, although they may have a limit on how much they are willing to jeopardize future reproduction [65]. Eggs from females that are in good condition during incubation and brood-rearing not only have high hatching success but also high fledgling success. This suggests that females, unlike males, do not compromise their body condition to achieve high reproductive success, probably because males hunt most prey for the brood [53] and therefore have a greater influence on reproductive success than females.

Experience in reproductive tasks, breeding habitat selection and familiarity with the surroundings can positively influence the reproductive performance of birds. Experienced, high-quality individuals are more likely to acquire and efficiently use good habitat [32,103,104], mate with a better compatible partner [23,105], start breeding early in the season [23,33] and have higher reproductive output [23,33,60], implying a high probability to produce a second annual brood [54]. In our study, experienced females had more eggs and a higher hatching success than inexperienced females. Experienced females may breed earlier [23,33] and therefore have a higher probability of producing a second annual clutch or produce larger first clutches than inexperienced females. In our study, experienced females were more likely to produce a second annual clutch in 3 of 4 years, suggesting that the observed effect is indeed very likely to be due to double brooding. While studies suggest that male birds of prey have no or only a weak effect on clutch size [106], our model suggests a negative correlation between experience and the number of eggs a male cared for per year. Experienced males cared for fewer eggs than inexperienced males, probably because experienced males may start breeding earlier in the season [23,33], when clutches tend to be smaller [60,65,92]. However, male clutch size seemed not to be linked with annual reproductive success, as we found no evidence that experienced males had fewer fledglings than inexperienced males.

Female reproductive performance appears to depend on her intrinsic quality but is probably also affected by the performance of her mate. High-quality males may enable females to breed earlier, achieve higher hatching success, maintain better body condition and produce more fledglings. When males provide sufficient food during incubation, females do not need to hunt and can consistently incubate, leading to higher hatching success [52] without compromising their own body condition [82,83,107]. Additionally, increased brood care from the male could enable the female to desert broods earlier and to initiate a second breeding attempt [54,108–110]. Reproducing with high-quality males would therefore result in high number of offspring under proportionally lower female workload possibly explaining the observed increased survival of females with an increasing number of produced fledglings. However, using mate experience as a broad proxy for quality, an initial modelling approach did not support this hypothesis. It would be interesting to investigate further how mate quality affects survival and reproduction, but this may require an additional linear predictor to model mate quality.

In our study, experienced males show higher survival than inexperienced males. The higher survival of experienced males can be attributed to the selective pressure exerted during their first winter and first reproduction. High-quality males are more likely to survive these critical phases of selection, leading to increased survival in experienced birds in subsequent years [111]. The lack of a relationship between experience and female survival might be due to the lower selective pressure of breeding on females. Males and females with larger eumelanin-based spot diameters showed a higher probability of survival than individuals with smaller eumelanin-based spots. That eumelanin coloration correlates with survival and thus represents an individual quality factor has been shown for several bird species [36,37,39,112]. In barn owls, the size of eumelanin-based spots has been consistently linked to coping abilities in stressful situations. Individuals with larger spots demonstrate better resilience in the face of food deprivation [27], increased resistance to oxidative stress [71] and a lower GC stress response during acute stress [70]. In addition, males with larger spots have been shown to be better providers when exposed to elevated GC levels [26], and females with larger spots reproduce earlier [39]. It has been suggested that elevated GC concentration in larger spotted females leads to a behaviour shift towards self-maintenance [58], which could lead to increased survival of larger spotted individuals under challenging conditions. In line with this, our data indicate that females with lower baseline GC levels and larger spots demonstrate higher survival rates. Therefore, larger spots in barn owls appear to serve as an indicator of individual quality.

## 6. Conclusion

Our study highlights the complex relationships between individual and environmental factors, reproductive investment and survival in barn owls. The results suggest that environmental and individual conditions can influence adult fitness through direct and indirect pathways. The strength of the associations between environmental and individual factors on survival and reproduction varies with sex and season. Prey availability determines the reproductive success of males and females, but high prey availability in winter is especially critical for male survival. Providing habitat with sufficient prey during the non-breeding period may therefore be of uttermost importance for male survival. In addition, males experience a distinct conflict between reproduction and survival, while female survival and reproduction depend mainly on the quality of the female and environmental conditions. The study therefore emphasizes the importance of considering multiple factors and including the different pathways in which they can be associated with fitness when examining reproductive success and its link to survival.

## Data Availability

Datasets and the R script to reproduce the analysis are available under: [113]. The habitat maps produced during the study are available from the corresponding author on reasonable request. Supplementary material is available online [114].
